# Tourist willingness to pay for local green hotel certification

**DOI:** 10.1371/journal.pone.0245953

**Published:** 2021-02-08

**Authors:** Katherine M. Nelson, Stefan Partelow, Moritz Stäbler, Sonya Graci, Marie Fujitani

**Affiliations:** 1 Leibniz Centre for Tropical Marine Research (ZMT), Bremen, Germany; 2 Ryerson University, Toronto, Ontario, Canada; 3 University of Bremen, Bremen, Germany; University of Maryland College Park, UNITED STATES

## Abstract

This study aims to understand tourists’ willingness to pay a price premium for a local green hotel certification, and is one of only a few in the literature for small-island tourism destinations in emerging economies with their unique and pressing sustainability challenges. In a survey of 535 tourists visiting Gili Trawangan, Indonesia, facing numerous waste management and coral reef conservation issues, the willingness to pay extra for sustainable hotel services was elicited. There were five discrete pricing levels across the surveys that ranged from $0.75 USD to $7.50 USD extra per night. We examined the relationship of the respondents’ payment choice to their socio-demographic attributes and attitudes regarding environmental issues such as climate change. The main findings and practical implications of the study are: (1) to demonstrate the broad willingness to pay for sustainable hotel services. Findings indicate at all price levels (between $0.75 USD and $7.50 USD), more than 50% of tourists are willing to pay. (2) To estimate a lower bound mean willingness to pay per night for a local green hotel certificate of $1.55USD and 1.34€ EUR, and (3) To identify individual attributes that influence willingness to pay. Findings indicate environmental knowledge and preferences play a role. These results can be used generally to incorporate evidence-based practices into the development of a green hotel marketing strategy, and to help define the target market for small-scale green hotel certification. Additionally, we propose a finance strategy for funding local and sustainable initiatives that support the hotel industry and the island’s infrastructure through the premiums collected from the ’Gili Green Award’ certificate.

## 1.0 Introduction

Concerns for social, economic and environmental sustainability have created demand for products and services in the tourism sector that can help achieve those goals. Tourism accounts for one in ten jobs globally, and ten percent of global GDP [1]. In 2017, there were more than 1.3 billion international tourists, up 7% from 2016. Since many tourists will stay overnight at their destination, hotels play a large role in the sustainability of the tourism economy [2]. Energy use and efficiency, cleaning and laundry products, water usage, waste and recycling practices, sewage management, single use products, packaging, transportation services, food and drink services, energy consumption and labor issues all contribute to a hotel’s environmental and social impacts [3].

Environmentally-friendly or ‘green’ certification schemes are emerging in the hotel sector because they signal the internal motivations (e.g., values, efficiencies, cost-savings) and external motivations (e.g., visibility, marketing) of hotels who aim to comply with environmental standards [4]. Although there are different motivations and strategies for complying with environmental standards [5], these efforts are an attempt to differentiate hotels on the market. Yet, environmental certifications differ in their criteria, and can be interpreted by tourists in different ways. This may confound the general notion of certification, with implications that influence guest satisfaction [6, 7]. Certifications provide tourists additional hotel selection criteria, but the costs of certification, if not internalized by hotels, are passed on to customers in the form of higher nightly prices [8]. In either case, understanding the value-added of a certification label is important for decision making [9].

The market viability of green hotel certifications, as a means to guide the tourism sector towards sustainability, is premised on tourists’ demand and willingness to pay for them. Therefore, to encourage hotels to engage in environmental responsibility measures (especially those that are not necessarily cost-saving), it must be clear that tourists care about environmental sustainability and this informs their hotel selection process. Numerous studies have examined whether people will pay (or pay more) for: environmentally friendly, eco-labeled or eco-certified products such as food [10–12] and furniture [13]; fair-trade food [14–16]; nature conservation [17–23]; eco-tourism services (e.g., tours and local cultural experiences) [24, 25]; socially responsible products [26] and carbon offsetting programs [27–29].

However, there is a limited literature examining tourists’ demand for environmentally responsible hotels, along with their willingness to pay, particularly within the context of a developing country such as Indonesia. One study by Peiró-Signes et al., [30] shows that guests tend to rate hotels with the ‘ISO 14001 international standard for sustainable operation’ higher than non-certified hotels, which informs the hypothesis that guests may also be willing to pay for certification. Chia-Jung and Pei-Chun [31] show which room items and hotel services influence green hotel choices among tourists in Taiwan and how much they are willing to pay, suggesting willingness may vary by what hotels offer beyond certification. This study builds on the existing few studies [31–36], to examine the preferences and factors driving potential variation among individuals in who is willing to pay higher prices for sustainability and how much.

It is estimated that over 140 eco-labels exist that focus on green hotel certifications, but only 6.2% of the hotels worldwide are certified, and in Asia only 0.9% of hotels [37, 38]. Despite many efforts to progress green hotel industry initiatives, the value of green certification to consumers is not well understood across contexts, which is perhaps reflected in the low percentage of hotels certified [39]. Given the total amount and diversity of tourists worldwide, and the hotel industry’s global contribution to greenhouse gas emissions, providing data to address the consumer valuation issue can be useful for both academic understanding to inform further studies and for industry in establishing certification schemes that meet consumer preferences and hotel economic models.

The multitude of eco-certification programs in the hotel industry and the criteria for certification which range from self-auditing, third-party regulation or hybrid approaches can be confusing for consumers and hoteliers alike. It is difficult to assess the key practices that contribute to the operation of a green hotel because there is no definitive regulatory body that governs eco-certification, although some databases exist (e.g., http://www.ecolabelindex.com/), leaving sustainability open to interpretation and compounding problems of industry ‘greenwashing’ which rightfully leads to consumer skepticism [40–42]. Creating detailed universal sustainability requirements that are applicable across the variety of hotel types and locations would fail to address context specific issues and practical implementation challenges. Well-known and established certification schemes may be appropriate for larger chain hotels and in more populated destinations, whereas locally developed and context-specific certification schemes may provide a better fit for popular tourist pockets in developing countries and small-island destinations. In either context, transparency is important as consumers demand information regarding environmental product attributes [43]. Additionally, the added value of green certification schemes is, presumably, that premium pricing can cover the additional costs of sourcing sustainable products / operational costs or the extra income can directly fund local conservation programs or support local groups [44]. The important factor for hotels is a focus on remaining competitive in a changing market while seeking a triple bottom line approach. This requires understanding the target market and creating innovative strategies that move beyond hotel industry norms (e.g., claiming a hotel is ’green’ based on standard practices such as asking guests to reuse linen and towels rather than having them changed daily).

It is obvious that a rise in the number of tourists and tourism infrastructure can have both positive and negative effects [45]. Improved general infrastructure, new sources of income, and the introduction of different cultures and life-styles of foreign visitors can generate positive outcomes to local people, but massive streams of tourists can be detrimental to local residents’ lives and cultures [46]. Moreover, tourism can lead to a degradation of nature, which in turn lowers the quality of life for residents and tourists [47, 48]. It is often difficult to assess and to handle the numerous side-effects associated with tourism, as markets for them are incomplete or nonexistent. These constitute externalities, benefits or costs that tourists or firms confer on others without paying or receiving compensation [49, 50]. Mechanisms to finance solutions for “internalizing” negative externalities from tourism include price-based measures (e.g., hotel taxes, tourist fees, voluntary contributions from businesses and tourists) and quantity-based measures (e.g., quota regulations) [23, 50]. Island economies face special challenges in achieving sustainable tourism [51], for example where regulation or government involvement in the coordination of environmental sustainability measures is lacking [52]. Therefore, self-financed and collectively coordinated programs that serve the interests of the community and local businesses may be the most viable option. Green hotel certifications have not been studied in this light before but they can provide a novel alternative to taxation to directly finance solutions to address negative tourism externalities.

In this study we examine the willingness to pay different price premium levels for a hypothetical local green hotel certification on the small but globally known tourism island of Gili Trawangan, Indonesia. We conducted a survey with a random sample of 535 tourists to elicit the willingness to pay a higher nightly price for hotels with a local green certification.

## 1.1 Coastal tourism and green hotels on Gili Trawangan, Indonesia

**Fig 1:**
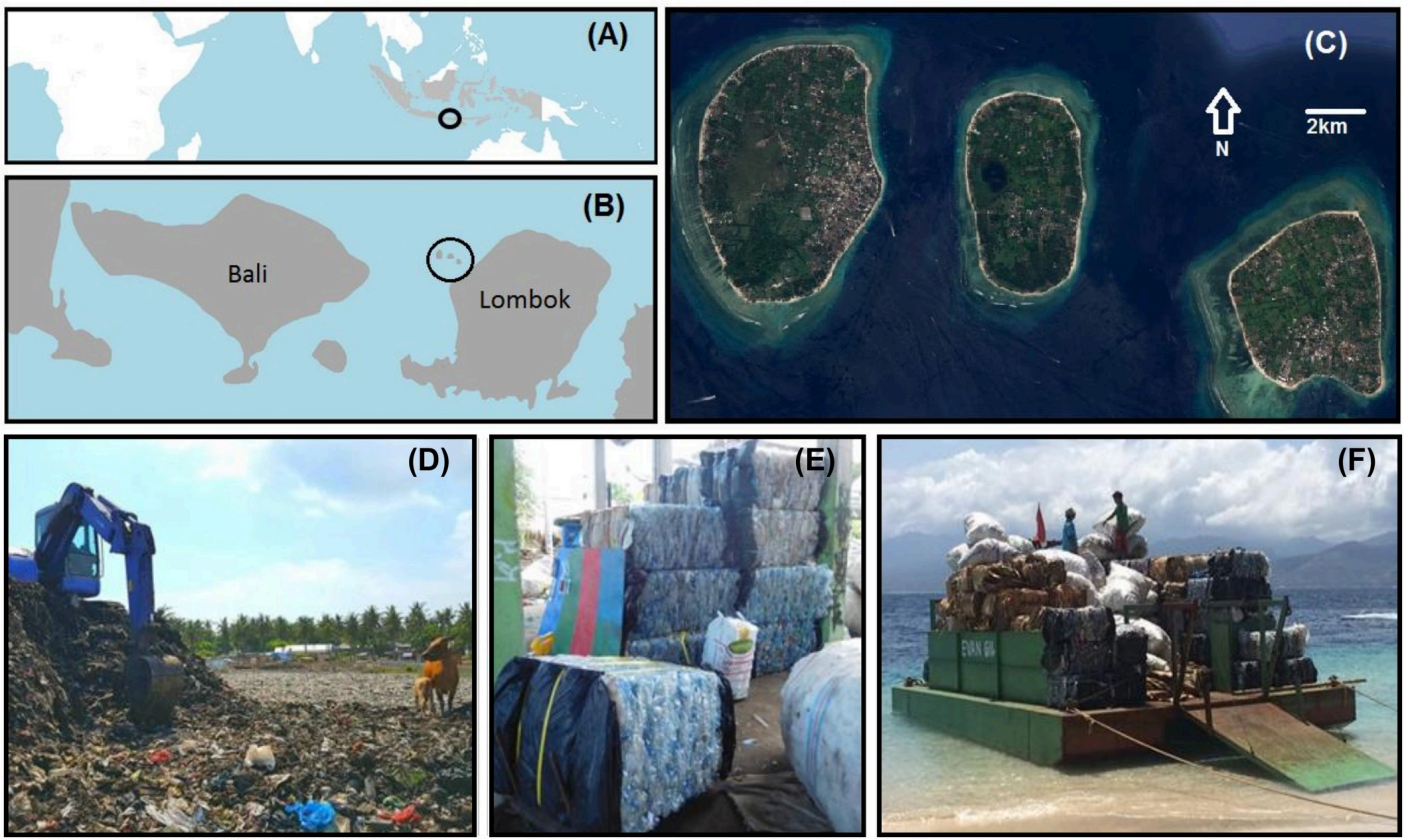
(A) Map of Indonesia with the location of the islands of Lombok and Bali circled. (B) The location of the Gili Islands with respect to Bali and Lombok. (C) Satellite photo of the Gili Islands. From left to right, Gili Trawangan, Gili Meno, and Gili Air. Source: Google Earth 2007. (D) Image of the landfill on Gili Trawangan. (E) Plastic bottles that have been sorted, prepared, and crushed for transport off the island by the Gili Eco Trust. (F) Gili Eco Trust transporting recyclable items off the island to the recycling center in Lombok.

Gili Trawangan is a six square mile island Bali and Lombok, Indonesia, easily reached by boat from both. Developing quickly since the 1980’s, it now receives up to a one million tourists a year, and has more than 45 SCUBA centers in the area. Accommodations on Gili Trawangan have 15 rooms on average and range from very small homestays to boutique-style hotels and villas and a few larger resort-style hotels, similar to other destinations such as Cancun, Aruba, or Phuket. There are no vehicles allowed on the island. The island faces numerous challenges for continued sustainable development including waste management and coastal/marine conservation [51, 53]. Tourism is the only economic activity on the island, as beyond subsistence agricultural and fishing activities, no goods (e.g., from manufacturing) are produced for sale. Everything must be shipped to cater to the thousands of foreign tourists daily. Until recently, virtually all waste, aside from refillable glass beer and soda bottles, remained on the island and ended up in the landfill, burned, or strewn around the island (see Fig 1).

The Gili EcoTrust is a local NGO that was established by dive shop owners and local business owners in 2000 to protect and restore the coral reefs from destructive fishing (http://giliecotrust.com/). It has grown into a multifaceted conservation organization that services all aspects of sustainability on the island. Financing comes through donations from local businesses, voluntary eco-payments from divers, volunteer labor, and through service contracts (i.e., for garbage collection services). With the money they organize garbage collection, recycling, community clean-up activities, make recycled bricks for construction, composting services and provide sorting training for hotel and restaurant staff. Good wages are ensured for garbage collectors and safe, sanitary working conditions (which did not exist before). The EcoTrust also collects, cleans, packs, and ships recyclable content off the island (see Fig 1).

An important recent effort includes the development of a local hotel certification scheme called the Gili Green Award. This will be a locally organized eco-certification for hotels based on compliance to various sustainability standards. Further details on the Green Award are provided below.

Many large international hotel chains and tourism companies are adopting more sustainable practices, however, it is still an emerging trend in developing states [54]. While international tourism firms may have access to best practices through parent companies, smaller tourism establishments face disadvantages associated with expertise, scale, and distance from suppliers, creating additional barriers for the adoption of new practices. Many companies, especially in the nature tourism sector, are also realizing that the reduction of negative ecological impacts protects the ecosystems and attractions on which their success is built [51]. However, sharing knowledge and adapting strategies to small-scale businesses on small islands in developing tourist destinations is not widely practiced. In such cases, locally organized sustainability programs may provide the most appropriate requirements that are tailored to the specific local conditions [23, 51, 53].

International sustainability requirements provide minimal guidance in the way of developing and meeting sustainable criteria for the small island destination of Gili Trawangan. The Gili Green Award partnered with sustainable development consultants (Go Green Bristol; http://www.businesswest.co.uk/gogreen) to define local sustainability requirements. The award is being designed as a locally organized eco-certification for hotels based on compliance to standards such as using biodegradable cleaners and packaging; installing energy-efficient light bulbs and electric equipment; avoiding single-use plastic products; tracking energy usage regularly; installing proper septic systems; sorting and recycling waste; reducing water use; educating staff and guests on sustainability; and reducing carbon emissions. Compliance to specific sustainability standards would warrant the use of the award label to verify that the hotel prioritizes eco-friendly practices. However, given the novelty of such certification for local tourism and in Indonesia, the value to consumers of a hotel eco-label is unclear. In order to encourage hotels to participate in the hotel certification program, the Gili EcoTrust expressed interest in conducting an explorative research study with tourists on the added value of an environmental hotel certification.

## 2.0 Methodology

This research received ethics approval from the host institute, the Leibniz Centre for Tropical Marine Research. All participation in the study was voluntary, informed consent was verbally obtained, and data were collected and analyzed anonymously.

### 2.1 Stated preferences–contingent valuation

Methods of non-market valuation are divided into two broad categories, stated preference or revealed preference. Stated preference methods are based on statements individuals make in response to questions about hypothetical situations. The individuals’ preferences are not observed but rather stated, whereas revealed preference methods rely on data that come from observation of people’s choices in real-world settings [55]. As revealed preference studies can only value existing goods, studies examining pricing for hypothetical goods are conducted using stated preference methods (including goods that don’t yet have an established market value such as new products/services or products entering into a new market) [15, 17, 19, 28, 56].

There are numerous data collection and analysis methods for examining stated preferences for eco-labeled goods. The survey-based contingent valuation method is the most commonly used and is well-accepted in the literature [55]. It should be noted that although stated preference methods have received criticism in the literature due to hypothetical bias, choice-based elicitation mechanisms are important as they may reduce this bias [57]. The National Oceanic and Atmospheric Administration (NOAA) panel recommends the use of a dichotomous choice [58, 59] format in contingent valuation surveys [60]. This format has several advantages: it is incentive-compatible, simple and cognitively manageable. The “take-it-or-leave-it” nature of dichotomous choice questions offer a single bid amount to which respondents choose ‘yes’ they are willing to pay or ‘no’, similar to real-world purchasing decisions [55, 61]. We have selected the dichotomous choice contingent valuation method and followed best practices during design and implementation; they are a well-accepted approach and present fewer problems than the open-ended and payment card alternatives, and they are subject to less anchoring bias than the double-bounded dichotomous choice format, for instance [55].

In the environmental literature, the willingness to pay a higher price for eco-labeled products has shown to be linked to pro-environmental values and attitudes [13, 33]. Demographic characteristics such as income levels, nationality, gender, and education may also influence price acceptability. Understanding the factors associated with willingness to pay can be used to better target specific audiences or to set stratified price structures based on these factors (e.g., different costs for the eco-label for budget, mid-range, and luxury hotel categories).

In one of the only studies examining the willingness to pay for green certified hotels, Kang et al., [33] show that customers with stronger environmental values are more willing to pay price premiums. They also show that customers staying at mid- or high-end hotels (also customers with higher income) are more likely to pay a price premium than those staying at budget hotels, and that male customers are more likely to accept higher prices than female customers.

### 2.2 Survey design

Together with the Gili EcoTrust, we designed and executed a survey eliciting tourists’ willingness to accept a nightly price premium for a hotel with the Gili Green Award environmental certification. Careful attention was paid during the design of the survey based on the fact that the value of the green award to consumers was not yet clear.

In order to design the bid structure for nightly price premiums for the survey, we drew on previous revealed preference donation studies conducted by the authors in Gili Trawangan [23, 62] and knowledge of other voluntary donations for environmental conservation solicited on the island. In addition, following the procedure described by Champ et al., [55], we conducted a pilot test with 50 randomly sampled tourists employing an open-ended question wherein participants were provided with the following scenario and question:

*Imagine you are booking your Gili Trawangan hotel on-line and there are two hotels that are identical in location*, *amenities*, *service*, *ratings*, *etc*. *However*, *one of the hotels has a ‘Gili Green Award*’* certifying it is an environmentally friendly hotel.

*How much additional would you be willing to pay per night for the hotel with the environmental certification*? *Rp*.*__________________________________*

*The ‘Gili Green Award’ *is an eco-certification for hotels based on compliance to standards such as using biodegradable cleaners*, *packaging*, *and energy-efficient light bulbs; avoiding single-use plastic products; tracking energy usage regularly; installing proper septic systems; sorting & recycling waste; educating staff and guests on sustainability; and reducing carbon emissions*.

The pilot test responses provided the basis for setting the range of price premiums. The price amounts selected for use in the survey were indicated in Indonesian Rupiah (IDR) with the equivalent values expressed in US dollars (USD) and Euros (EUR): 10,000IDR ($0.75USD, 0,70€EUR), 20,000IDR ($1.50USD, 1,40€EUR), 50,000IDR ($3.75USD, 3,50€EUR), 70,000IDR ($5.25USD, 4,90€EUR), and 100,000IDR ($7.50USD, 7,00€EUR) extra per night of hotel stay. For reference, at the time the average price range of accommodations on Gili Trawangan was $20.00USD (for single/homestay/hostel) to $40.00USD (for double hotel room) per night [63]. Monetary sums discussed in this paper reflect the exchange rates on May 17, 2017 (i.e., 1 USD = 13,327.94 IDR; 1 EUR = 14,881.38IDR. Source: https://www.exchange-rates.org/HistoricalRates/A/IDR/5-17-2017)

The survey instrument (Appendix 1 in S1 File) included demographic questions (e.g., age, nationality, gender, income), as well as questions about their visit to the island (e.g., hotel name, number of days on island, perceptions of environmental sustainability of hotels/businesses on island), and their environmental beliefs (e.g., climate change understanding). Questions regarding environmental beliefs targeted awareness of consequences and ascription of responsibility towards climate change, as well as self-reported knowledge, as these are relevant to understand motivations of pro-environmental behavior [64–66]. Items were adapted from the Yale Environment Poll, Resources for the Future, and the Pew Research Center (Appendix 2 in S1 File). To elicit the acceptability of the price premium level for the green hotel award, each survey included a dichotomous choice (‘yes’/’no’) question. The discrete price level indicated on each of the surveys was randomized across the sample. The following is an example of the wording for the price premium question for the 10,000IDR price level:

*Imagine you are booking your Gili Trawangan hotel on-line and there are two hotels that are identical in location*, *amenities*, *service*, *ratings*, *etc*. *However*, *one of the hotels has a ‘Gili Green Award*’ *and costs Rp*. *10*.*000 more per night (about $0*.*75USD / 0*.*70€ EUR)*.

*Would you book it*? ***YES NO***

*(Please answer honestly and keep your current budget in mind*)

In the survey we provided the explanation of the Gili Green Award to tourists, defined as an eco-certification for hotels based on compliance to standards described above in section 1.1. After the payment question, respondents were followed up with, to identify protest responses as well as the rationale for those who said they were not willing to pay.

### 2.3 Sampling

We surveyed 535 tourists from May 11 to June 7, 2017. Tourists were randomly sampled (i.e., no preferential selection criteria among all tourists) from the harbor area between 10am and 3pm daily while waiting for their ferry to depart the island, the only way and place to get on and off the island. This area provides a representative population of tourists given that tourists congregate here to depart the island whether by public ferry or private boat. The overwhelming majority of tourists depart from the public harbor area between 10am and 3pm daily, aside from a small percentage (~1%) that arrange individual speed boat transfers departing directly from the hotel. Participants were approached by field assistants that were volunteer interns for the Gili EcoTrust and were asked if they could fill out a short survey. Participation was entirely voluntary, verbal informed consent was obtained from all respondents, and all data were collected anonymously. Participants were given a short reminder to consider their current budget and to answer honestly as if they would be asked this question by their hotel. All surveys were in English and were printed on the front and back of a half sheet of A4 paper. English is widely spoken on Gili Trawangan and is the most common method of communication with tourists on the island. Field assistants also spoke French, German, and Bahasa Indonesian and would help translate when needed. The surveys were mixed so that the treatments were randomly allocated to participants.

### 2.4 Data analysis

Within the 535 completed questionnaires, three respondents refused to answer the dichotomous choice question at all and were dropped from the analysis, and a further nine respondents were deemed protest bids and excluded from further analysis. The nine protest bids were defined based on their conflicting responses to the payment question (marked “yes”) and the conditional follow-up question that should only be answered if the response to the payment question was “no”. Nine respondents indicated “yes” they would pay the Gili Green Award premium and then also responded with reasons why they would not pay the premium. Therefore, it is clear that the respondents were either not paying attention or were purposely marking contradictory information in an act of protest for an unknown reason, and thus, they were dropped from analysis. Statistical analysis was conducted in R [67].

To identify the characteristics of respondents associated with willingness to pay, we fit a logistic regression model to the data (N = 526). In a generalized linear model (GLM) with binomial error distribution and logistic link function [68], we modeled the likelihood to accept the payment level for the Gili Green Award as a function of demographic and environmental attitude variables:

$$\mathrm{Prob}\;(Y|X)={\left[1+\exp\left(X_{p}^{\prime c}\alpha_{glm}+X_{q}^{\prime d}\beta_{glm}+{\tilde{X}}_{r}^{\prime d}{\tilde{\beta }}_{glm}\right)\right]}^{-1}$$

where *Y* is the dependent dichotomous factor for the payment level *Y* = 0 or 1, *X* = (*X*^*c*^, *X*^*d*^*, ${\tilde{X}}^{d}$*) denotes a vector of *p* integer (age, and its quadratic effect, and days spent on the island), *q* discrete unordered (gender, World Bank region, perception of human role in climate change, opinion of who should assume financial responsibility for climate change and of the necessity to change individual lifestyles to mitigate climate change) and *r* discrete ordered (hotel category, perceived income, understanding of climate change issues and beliefs in governmental agency) explanatory components, and the α’s and β’s represent parameters associated to the related regressors. Interactions between the explanatory terms were not considered. The model was reduced to the minimal adequate model by stepwise removal of non-significant terms [68]. With the final model established, the model’s compliance with assumptions and performance were assessed and its main effects investigated.

The acceptance rate, as the ratio of positive to total responses, was calculated for the overall sample, and per payment level. Lower bound mean willingness to pay was calculated using the non-parametric Turnbull estimator [69]. The Turnbull estimator was chosen as it makes no distributional assumptions about willingness to pay, unlike parametric estimators of central tendencies, and constrains the probability of willingness to pay to positive values that sum to unity across bid intervals. A lower bound estimate was selected as the conservative figure so as to not overestimate willingness to pay.

## 3.0 Results

### 3.1 Descriptive statistics

Table 1 shows the descriptive characteristics of the overall sample and for each of the payment levels. The sample sizes are relatively even across the payment levels and there are no statistically significant differences in the descriptive characteristics across the groups using a Pearson’s Chi^2^ test. The average age of participants was 28–29 years. On average there were slightly more females that participated than males. Participants stayed on the island for an average of about 4 days and reported their income to be ‘below average’ relative to others in their country. As such, hotel stayed at is taken to be a better measure of budget constraint than self-reported income level, as it provides us the amount actually paid, though the respondents’ perceived relative income remains informative. For analysis, countries of origin were grouped according to the regions defined by the World Bank. The overwhelming majority (>75%) of tourists came from a European country. These statistics are in line with previous tourism studies in the region showing that our sample captured a representative population of tourists on Gili Trawangan [23].

**Table 1:** Descriptive statistics of the sample population.

	Overall	Payment levels (IDR)				
		10,000	20,000	50,000	70,000	100,000
*N*	535	108	106	107	107	107
Average Age	28.5	28.4	28.3	28.8	28.1	29
Female	55%	57%	61%	52%	52%	54%
Average # days on island	4.1	4.0	4.0	3.7	4.7	4.0
Income	2.0	2.1	2.1	1.9	2.0	2.0
European & Central Asian	75%	79%	75%	76%	76%	75%
Latin American & Caribbean	5%	1%	9%	4%	3%	8%
Middle East & North Africa	1%	1%	2%	0%	2%	0%
North America	7%	4%	5%	10%	9%	8%
South Asia	0.5%	1%	0%	0%	0%	2%
Sub-Saharan Africa	0.3%	0%	1%	0%	0%	1%
South East Asia & Pacific	11.5%	14%	8.5%	9%	11%	7%

Note: Income is measured on a 5 point Likert scale with the range from 1 ‘Far below average’, 2 ‘Below average’, 3 ‘Average’, ‘Above average’, and 5 ‘Far above average’.

### 3.2 Statistical analysis

**Fig 2:**
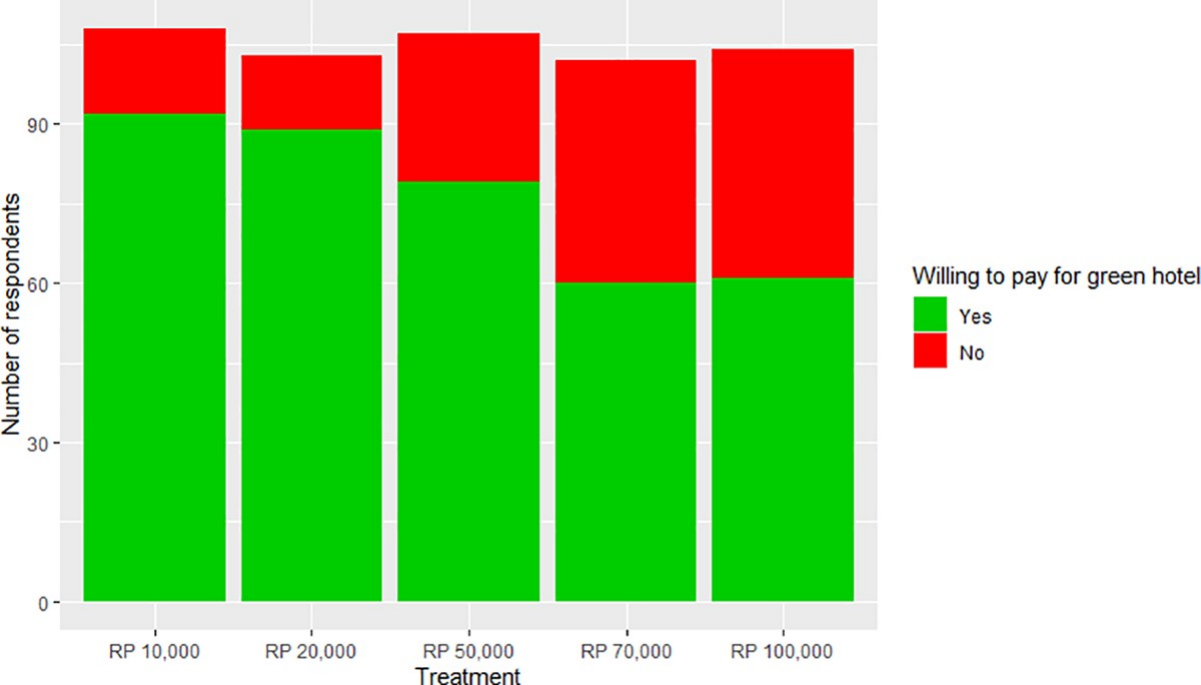
Acceptance of payment for the ‘Gili Green Award’ declines significantly with the amount requested extra per night (‘Treatment’, p < 0.001).

On average 72.7% of tourists were willing to pay extra for a green labeled hotel. Positive responses decreased with increasing amounts requested (p<0.001), consistent with economic theory, with payment levels 10,000IDR and 20,000IDR leading to 85% and 86% acceptance rates, and 70,000IDR and 100,000IDR both to 59% acceptance (Fig 2).

**Fig 3:**
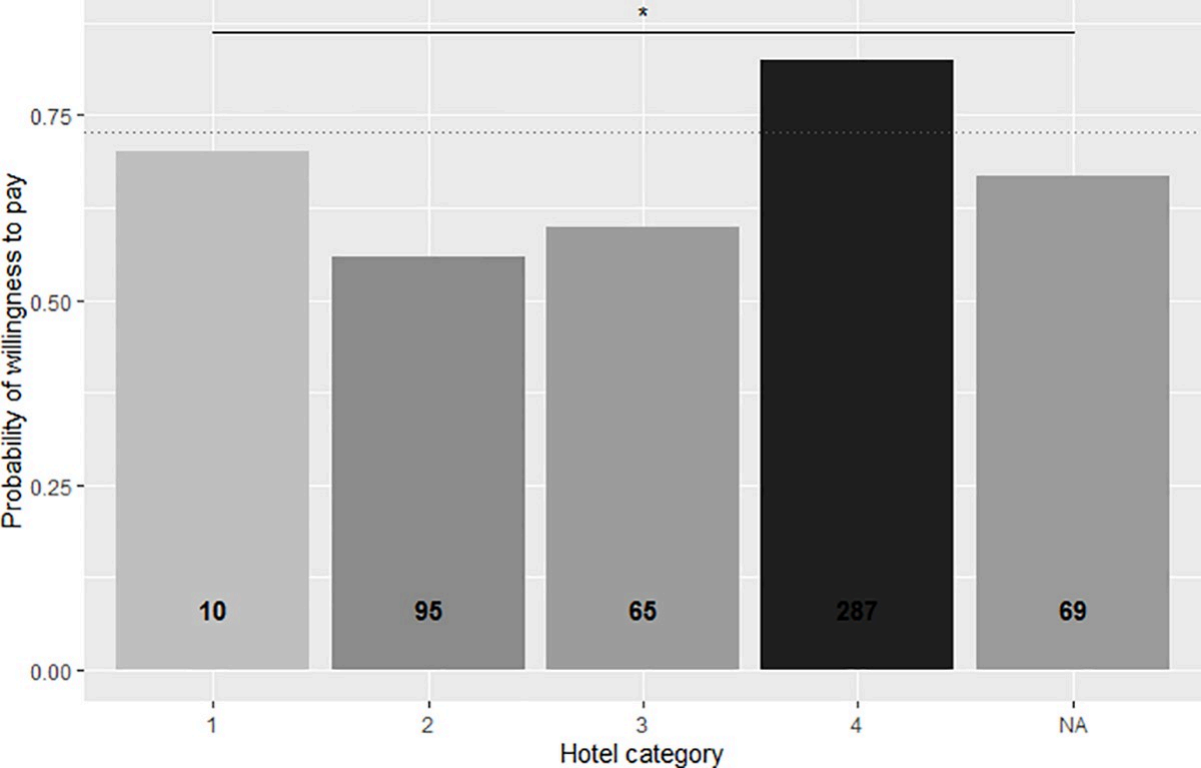
Propensity of respondents to pay the price premium indicated for the Gili Green Award as a function of the category of the hotel where they stayed. Note: Hotels categorized as ‘1’ if <120,000IDR/night; ‘2’ if 120,000–200,000/night; ‘3’ if 200,000–400,000/night; ‘4’ if >400,000IDR/night. Shading and numbers on the columns indicate the respective numbers of respondents in each category.

**Fig 4:**
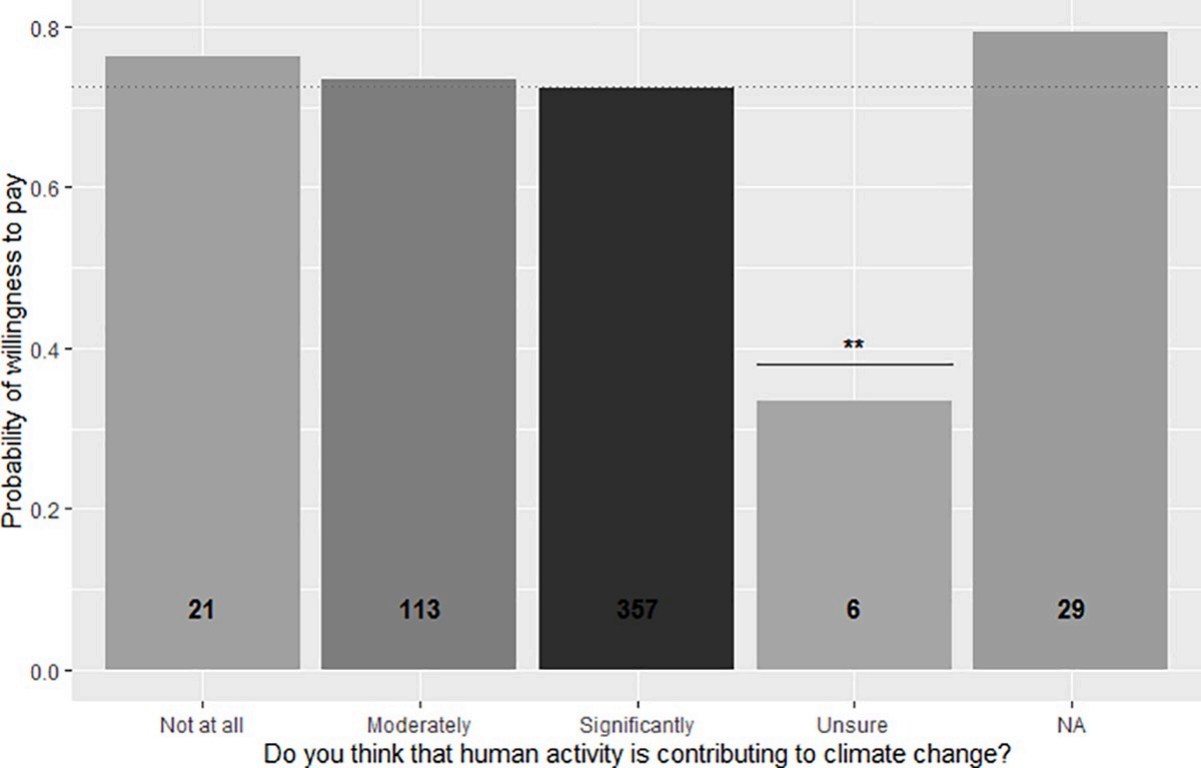
Propensity of respondents to pay the price premium for the Gili Green Award based on participants’ opinion of the contribution of human activity to climate change. Shading and numbers on the columns indicate the respective numbers of respondents in each category.

**Fig 5:**
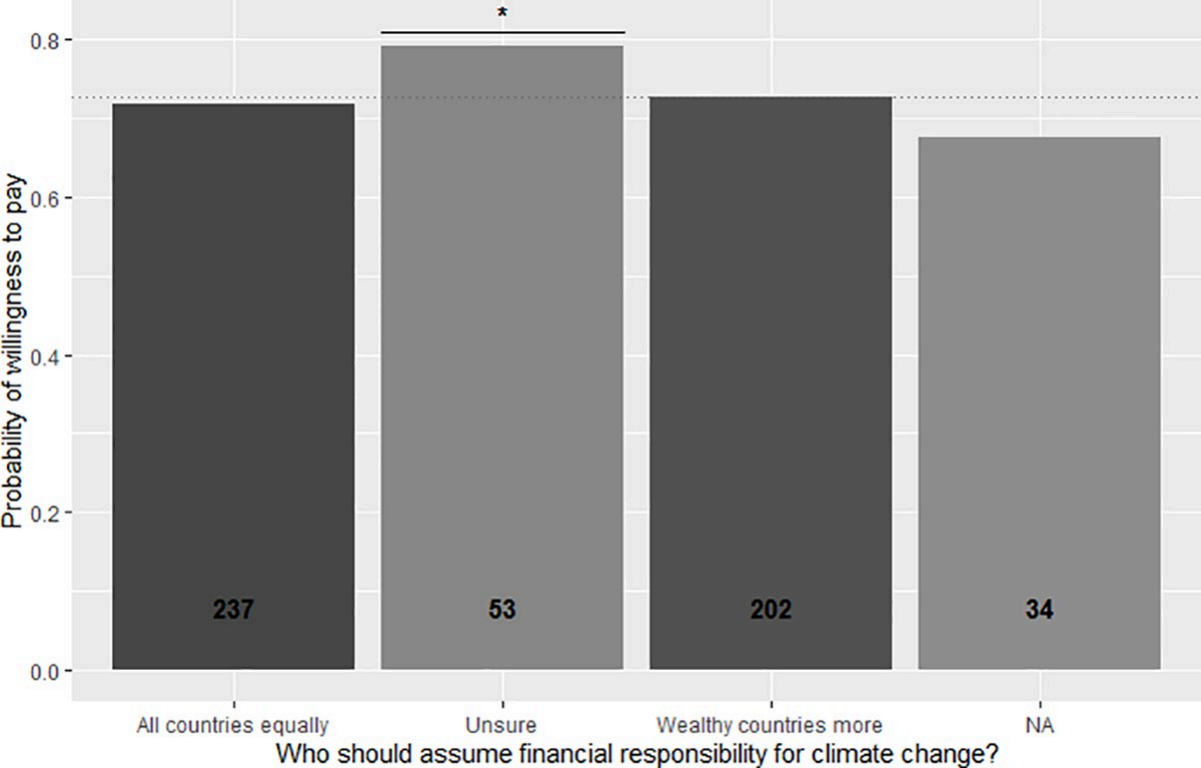
Propensity of respondents to pay the price premium for the Gili Green Award based on participants’ opinion about how financial responsibility for climate change should be divided across countries. Shading and numbers on the columns indicate the respective numbers of respondents in each category.

**Fig 6:**
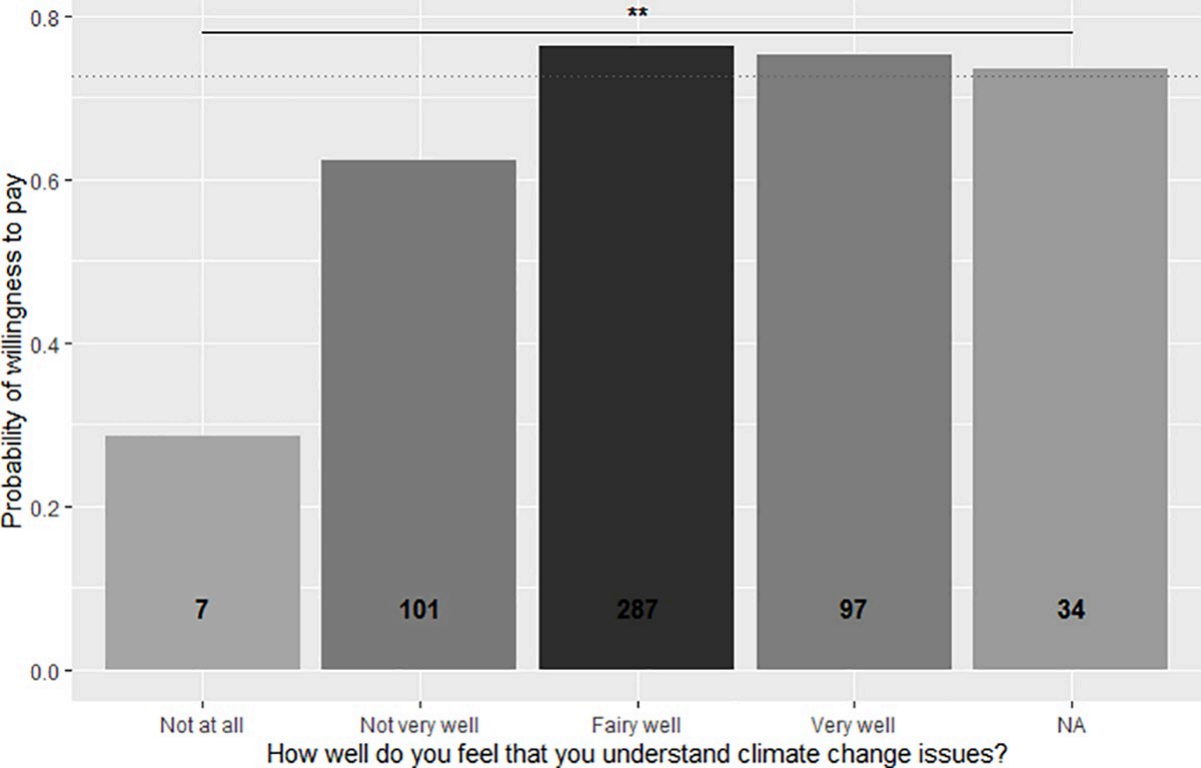
Propensity of respondents to pay the price premium for the Gili Green Award based on participants’ opinion of how well they claim to understand climate change issues. Shading and numbers on the columns indicate the respective numbers of respondents in each category.

Although the acceptance rate decreases as the price of the Gili Green Award increases, this drop levels off at 70,000IDR and remains constant at the 100,000IDR price. The full GLM model (Akaike’s Information Criterion (AIC) = 381.74) utilized to identify personal traits significantly associated to respondents’ willingness to pay was reduced to the minimal adequate model by stepwise removal of non-significant terms. All explanatory factors significantly associated with the willingness to accept the payment level are listed in Table 2, together with their effect on the logarithm of the odds of being likely to accept the payment level (‘Estimate’), their standard error, and associated p-value. The significant variables from the GLM model are explored in more detail in individual graphs in Figs 3–6.

**Table 2:** Coefficients of the final model of the logistic regression of factors associated with the log odds of guests’ willingness to pay extra for hotels carrying the Gili Green Award label.

	Estimate	Standard Error	z value	Pr(>|z|)	
(Intercept)	-1.496	1.241	-1.205	0.228	
Treatment (Price premium)	-2.096x10^-05^	-3.932x10^-06^	-5.331	0.000	***
Hotel category	0.653	0.530	1.232	0.218	
Hotel category (Quadratic)	1.081	0.437	2.472	0.013	*
Hotel category (Cubic)	-0.316	0.315	-1.001	0.317	
Human activity: Not at all caused by humans	3.030	1.378	2.199	0.028	*
Human activity: Moderately caused by humans	3.761	1.293	2.908	0.004	**
Human activity: Significantly caused by humans	3.649	1.275	2.863	0.004	**
How to fund CC: Equal responsibility across all countries	-1.132	0.505	-2.240	0.025	*
How to fund CC: Wealthy countries should pay more	-0.940	0.513	-1.835	0.066	.
Understand CC.	2.176	0.866	2.514	0.012	*
Understand CC. (Quadratic)	-0.847	0.654	-1.295	0.195	
Understand CC. (Cubic)	-0.124	0.350	-0.353	0.724	

Significance codes

‘***’ p<0.001

‘**’p<0.01

‘*’p< 0.05

‘°’ p<0.1

Reference categories for the ordered factors are the lowest category (i.e., Hotel category = 1; Understand CC. = “Not at all.” Unordered factors (Human activity and How to fund CC) have”Unsure” as the reference category.

Note: Indicators obtained from questionnaire. Variables: ‘Treatment’ *(Accepted Price Premium of Gili Green Award*, *10/20/50/70/100 = 10*,*000IDR/20*,*000IDR/50*,*00IDR/70*,*000IDR/100*,*000IDR); Age (integer)*, *Hotel Category (Categorical*: *‘1’ <120*,*000IDR/night; ‘2’ = 120*,*000–200*,*000IDR/night; ‘3’ 200*,*000–400*,*000IR/night; ‘4’ >400*,*000IDR/night; Country regions grouped according to World Bank Regions (Categorical); Climate change caused by humans (Categorical*: *1 = Significantly 2 = Moderately 3 = Not at all 4 = Unsure); Climate funding (Categorical*: *1 = Equal responsibility 2 = Wealthy countries pay more 3 = Unsure); Understand climate change (Categorical*: *Scale from 1 ‘very well’ to 4 ‘not at all’;*.

The price coefficient is negative and very small, due to the units of currency (IDR). The log odds of respondents’ likelihood to accept the price premium rate increased quadratically with hotel category (Table 2, Fig 3). As such, visitors’ propensity to pay increased non-linearly with increasing level of hotel category. Those staying in hotels costing more than 400,000 IDR per night (hotel category ‘4’) were most willing to pay higher price premiums. Also, this was the most commonly represented hotel category by far.

Respondents expressing a range of opinions about human activity and its relation to climate change had a significantly higher propensity to pay a price premium than those that indicated they were unsure about the influence of human activity on climate change. However, and interestingly, the content of the respondents’ distinct opinions (ranging from human activity significantly causing climate change or not at all) did not lead to significant differences in their probability to pay for the Gili Green Award (Table 2, Fig 4).

In similar fashion, only significant separation was found between interviewees that claimed to be unsure about where financial responsibilities for climate change should lie, as opposed to those believing they should be assumed by all countries equally, or by wealthy states in particular. Those that did express an opinion were found less likely to be willing to pay for a hotel with a green certification. For those believing wealthy countries should pay more, the negative effect on willingness to pay is significantly different from those unsure only at the p < 0.1 level (Table 2, Fig 5). Neither advocates of equal burdens, nor those wanting those to be weighed by wealth, deviated from the population’s average behavior.

With increasing (perceived) understanding of climate change along the ordered factor from ’not at all’ to ’very well’, the probability of interviewees being willing to pay increases linearly (Table 2, Fig 6). The better visitors felt they understand climate change processes, the more likely they were to accept the price premium for the green hotel label.

The Turnbull nonparametric estimate of lower bound mean willingness to pay per night for a hotel with a green certification across the sample was 22,285 IDR, or about $1.55USD and 1.34€ EUR. Based on summary statistics and model results showing the importance of hotel price category, lower bound mean willingness to pay was also analyzed based on reported hotel price category where available, combining categories 1–3 (up to 400,000 IDR a night; n = 172), and category 4 (greater than 400,000 IDR a night, n = 290). For respondents staying in hotels of price categories 1–3 (nightly rates up to $27.70USD or 24.04€ EUR) the lower bound mean willingness to pay was 14,826 IDR (about $1.03USD or 0.89€ EUR), and for hotel price category 4, with nightly rates greater than the prices mentioned above, the per-night lower bound mean willingness to pay was 23,724 IDR (about $1.64USD or 1.43€ EUR).

## 4.0 Discussion

At all price levels of this study (between $0.75 USD and $7.50 USD), more than 50% of tourists indicated willingness to pay a price premium for a hotel certified with the Gili Green Award. More than 80% probability to accept the rate was indicated at the lowest price levels ($0.75-$1.50 USD extra per night). This contrasts previous studies such as Kang et al., [33] showing only a 29% willingness to pay among tourists in Florida, Arizona and Texas although they did not provide price levels, they simply asked if they were willing to pay more. This can also be attributed to the fact that in the Kang et. al., [33] study most respondents were from a different geographic area and were also a different type of tourist. Similarly, López-Sánchez and Pulido-Fernández [34] show that 26% of the tourists surveyed in Spain had a positive willingness to pay, though they also did not calculate an estimate. Again, this low amount could be attributed to the cost of accommodations to begin with and also to the different types of tourists in the destination. Thus, our study suggests that the proportion of participants willing to pay for a green certified hotel on Gili Trawangan is substantially higher than the existing studies in the literature. We attribute this effect to the fact that the small island setting allows visitors to reflect on their personal impact on the environment as they face the boats ferrying thousands of visitors and goods to the island daily and they see the accumulation of garbage on the streets, beaches, in the ocean, and in the highly visible overflowing landfill in the middle of the island. It is not possible to hide the impact of humans on the environment in such a setting. This highlights the important of coupling willingness to pay studies with inquiries into how and why this varies by context, either at the local destination or among the tourist population visiting. This is good example of how pairing quantitative experimental work with richer inquiries about context and perception through qualitative interviews can increased the ability to explain findings with more certainty. Prior environmental knowledge, relative purchasing power, activities engaged in and island size can all be important explanatory factors pursed by multiple methods.

Additionally, a large majority of tourists on Gili Trawangan travel from far distances to enjoy this island, and, as climate change rhetoric becomes more common in daily life, people are more aware of the impact of such long-distance travel on their global environmental footprint and may be more willing to pay to offset some of the impacts [27–29]. The fact that most respondents were willing to accept the price premium is also reflective of the cost of accommodations and the fact that the young age of many of the tourists reflect their knowledge about sustainability. In the United States and Spain there are varied socio-demographics amongst the tourists studied as well as higher cost for the accommodations provided. Willingness to accept price premiums for environmentally certified goods may be influenced by the context and/ or socio-demographics and environmental preferences of the tourist population, as well as, the method of asking for the environmental payment [23, 70].

Further, even among the few studies in the literature exploring willingness to pay for green hotel initiatives, most only explore factors influencing willingness to pay more [8, 33, 34]; ours is one of the only to calculate a mean willingness to pay for a price premium associated with a green hotel certification. We estimate the lower bound mean willingness to pay per night is 22,285 IDR, or about $1.55USD and 1.34€ EUR. A contribution of this magnitude seems small, but given the over one million tourists visiting Gili Trawangan a year, and the average vacation length on the island of about four nights, this adds up to a substantial sum. Though an aggregated welfare estimate is beyond the scope of this study, we show the potential of the Gili Green Award to both generate revenue to support sustainable tourism as well as provide hotels with means to signal and fund their commitment to sustainability. There is an abundance of accommodation options resulting in high competition between hotels on Gili Trawangan (e.g., Tripadvisor.com shows 549 accommodation properties). With such high competition between hotel options, the hotels are understandably reluctant to increase prices for fear of losing business to hotels offering lower prices. Green hotel certificates can benefit hotels by providing market differentiation from other similar hotels which can attract customers. Websites such as TripAdvisor and Hotels do identify hotels that have been certified as green and this may be able to provide benefit to participant hotels.

Although it is clear from our study that many visitors would be willing to pay for green hotel certification, and sustainability is important to them, several demographic and attitudinal variables provide further segmentation that could be useful to target specific groups based on their preferences. Hotels could use this information to market sustainability certification to travelers that meet these criteria, especially if such travelers are more likely to book through travel agencies or group tours where green certification can be featured in marketing material. Likewise, demand for hotel sustainability information from consumers may lead to further on-line travel booking sites providing filters for sustainability criteria and green certification, although standardization of certification is important to avoid ‘greenwashing’. A market segment that are willing to pay higher prices for the Gili Green Award includes those staying at hotels that cost more than 400,000IDR per night. Thus, a progressive price premium could be used, that scales with the with the price of the hotel. However, there is potentially a low ceiling to the progressive rate, as the mean willingness to pay calculated for this segment is about $1.64USD or 1.43€ EUR. Our results suggest that for hotels costing up to 400,000IDR per night ($27.70USD or 24.04€ EUR) a contribution towards the Gili Green Award of $1.03USD or 0.89€ EUR would be suggested.

Further, this study elucidates the relationship between the respondents’ willingness-to-pay for sustainability labels and their perception of who is responsible for degrading the environment. Information from this study can help frame the marketing communication strategy that may differ between market segments, for example around local or global sustainability. Framing sustainability issues around local pollution may be important for communicating to those who do not believe their actions have a role in global climate change compared to framing sustainability around global climate change to those that respond more strongly to humans’ role in climate change. Given that those respondents who do not believe human activity contributes to climate change (N = 21) or those who believe that humans only moderately contribute to climate change (N = 113) still overwhelmingly (>70%) accept the price premium for the Green Award provides valuable information on the importance of framing sustainability as a local issue (i.e., reducing water use and waste vs. reducing the impact of your emissions from traveling/offset carbon footprint).

Based on the similar concept of offsetting one’s personal environmental footprint through carbon offsetting when purchasing air/bus travel, a financing model for the Gili Green Award in which additional revenue coming from tourists paying a green premium could go to the Gili EcoTrust to source bulk sustainable products for hotels and to support their environmental management initiatives on the island may help. This could lead to hotels being rewarded for compliance with more customers and increased reputation. The funds raised could also go towards management of the Gili Green Award, the Gili EcoTrust and training for all the hotels on the island to learn about initiatives that can be implemented to increase sustainability. Building capacity and knowledge in terms of sustainability amongst the hotel owners on the island would lead to a successful implementation of the Gili Green Award that would also support continuous improvement in this sector. Our findings suggest that the price premiums would be most effective if presented as a default opt-out, and added to the nightly stay of the tourist during booking, where effort would be needed in order not to participate rather to participate. Although the mean willingness to pay is low ($1.55USD or 1.34€ EUR), this is a lower bound conservative estimate. When aggregated across the number of tourists staying across all potentially certified hotels, it becomes a substantial amount. Overall, given the high acceptance rates, the opt-out option would lead to a smoother uptake of the increase than a voluntary opt-in, as has been experimentally verified on Gili Trawangan [23].

It is important to note that any additional costs to tourists for hotels, could crowd out spending for other things (e.g., they eat out less or dive less due to increased hotel costs). However, we believe our price levels are below the threshold where this would occur. If a tourist perceives this trade-off, then they may select a hotel at lower price levels that also includes the fee. If they do choose to pay the fee over other activities, we can ask, is that a bad thing overall? From one perspective, they would then be contributing to financing pro-environmental efforts on the island with their visit, the environment that the local economy depends on. On the other hand, they may not be supporting local businesses directly, although by doing those activities (e.g., diving), they might be increasing the environmental impact of their visit. These are important questions for future research, to consider any practical economic and environmental effects that may play out from decision-making processes after implementation.

## 5.0 Conclusion

Findings indicate that at all price levels (between $0.75 USD and $7.50 USD), more than 50% of tourists are willing to pay. The estimate of lower bound mean willingness to pay per night for a hotel with a green certification across the sample was 22,285 IDR, or about $1.55USD and 1.34€ EUR. We suggest an opt-out and selected price level choice architecture to generate the most revenue while maintaining high acceptance rates among tourists. However, acceptance rates are influenced by tourist environmental knowledge, hotel preferences and demographics.

Further studies on testing a pricing stratification should be conducted as well as to determine what specific factors or initiatives tourists find important to support when it comes to sustainability. Perceptions and knowledge of existing environmental issues are important factors to help unpack the reasons behind why tourists are willing to pay or not. These are important to understand to increase contributions by adapting choice architecture in ways that appeals to tourist perceptions of important factors and existing public knowledge. In general, there is a need in the tourism industry to find sources of funding to support sustainability initiatives that are driven by consumer demand and allow that demand to be reflected in available market choices that hold businesses accountable to values, and our study provides findings indicating that incorporating price premiums into tourism products through certifications such as accommodations is one way to ensure that financing is provided through a mechanism and price level that a large majority of tourists find acceptable and willing to support, while still maintaining the freedom of choice.

## Supporting information

S1 File (DOCX)
