# Supplementary material for: Tourist willingness to pay for local green hotel certification
Source: PLoS One. 2021 Feb 8;16(2):e0245953. doi: 10.1371/journal.pone.0245953 (PMC7870074; doi:10.1371/journal.pone.0245953)
Supplement: S1 File — (DOCX) [file pone.0245953.s001.docx]

**Appendix 1. Sample Instrument**


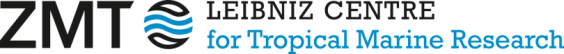

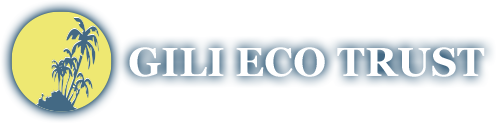


We hope you enjoyed your stay on the island! The purpose of this survey is to understand tourist perceptions and it will be used to help us continually improve the sustainability of Gili Trawangan. We greatly appreciate your feedback!

| Age: | Nationality: | | | | Gender: M F | | |
| --- | --- | --- | --- | --- | --- | --- | --- |
| Number of days on island: | | Name of hotel: | | | | | |
| More businesses on Gili T should adopt environmentally friendly practices. | | | Strongly Disagree | Disagree | | Agree | Strongly Agree |
| I am willing to pay higher prices to support businesses and products that are environmentally conscious. | | | Strongly Disagree | Disagree | | Agree | Strongly Agree |
| Imagine you are booking your Gili Trawangan hotel on-line and there are two hotels that are identical in location, amenities, service, ratings, etc. However, one of the hotels has a ‘Gili Green Award’* and costs Rp. 100.000 more per night (about $7.50USD/7.00€).  Would you book it? **❑ YES ❑ NO**  (Please answer honestly and keep your current budget in mind) | | | | | | | |
| If you answered no, why? Check all that apply:  **❑**I don’t care about green certifications. **❑**This amount is too high, I would pay less. **❑**It’s a good idea but I’m not willing to pay extra for it.  **❑**If I had more money, I would pay for it. **❑**I don’t believe it makes any difference. **❑**The extra money doesn’t go towards what is proposed. | | | | | | | |

*The ‘Gili Green Award’ is an eco-certification for hotels based on compliance to standards such as using biodegradable cleaners, packaging, and energy-efficient light bulbs; avoiding single-use plastic products; tracking energy usage regularly; installing proper septic systems; sorting & recycling waste; educating staff and guests on sustainability; and reducing carbon emissions.

| What is your income status relative to others where you come from? | **Far below average** | **Below average** | **Average** | **Above average** | **Far above average** |
| --- | --- | --- | --- | --- | --- |
| 1. Do you think that human activity is contributing to climate change? ❑Significantly caused by humans ❑Moderately caused by humans ❑Not at all caused by humans ❑Unsure | | | | | |
| 2. Who should assume financial responsibility for climate change?❑Equal responsibility across all countries ❑Wealthy countries should pay more ❑Unsure | | | | | |
| 3. How well do you feel that you understand climate change issues?  ❑Very well ❑Fairly well ❑Not very well ❑Not at all | | | | | |
| 4. How much do you think government regulations designed to reduce global warming will help to curb the warming? ❑Significantly ❑Somewhat ❑Not too much ❑Not at all | | | | | |
| 5. Global climate change can be reduced:❑only if individuals make major lifestyle changes ❑even if individuals do not make major lifestyle changes❑it’s not possible to reduce climate change ❑unsure | | | | | |

**Appendix 2. Question sources for environmental awareness and climate change beliefs**

1. Do you think that human activity is contributing to climate change?

The Yale AP-NORC Environment Poll 2018

<https://climatecommunication.yale.edu/about/projects/environment-poll/>

2. Who should assume financial responsibility for climate change?

Pew Research Center Spring 2015 Global Attitudes Survey

https://www.pewresearch.org/global/2015/11/05/2-public-support-for-action-on-climate-change/

3. How well do you feel that you understand climate change issues?

The Yale AP-NORC Environment Poll 2018

<https://climatecommunication.yale.edu/about/projects/environment-poll/>

4. How much do you think government regulations designed to reduce global warming will help to curb the warming?

Resources for the Future Public Attitudes on Global Warming 2018

<https://www.rff.org/energy-and-climate/surveying-american-attitudes-toward-climate-change-and-clean-energy/>

5. Global climate change can be reduced if:

Pew Research Center Spring 2015 Global Attitudes Survey

https://www.pewresearch.org/global/2015/11/05/2-public-support-for-action-on-climate-change/

**Appendix 3. Log odds of willingness to accept the payment level based on age of participants**


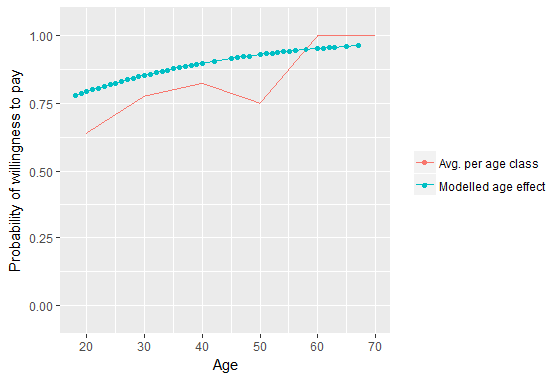


Probability of accepting the payment level for the Gili Green Award increased linearly with age, hence its probability rose in a logistic manner
